# Effect of Filler Alignment on Piezo-Resistive and Mechanical Properties of Carbon Nanotube Composites

**DOI:** 10.3390/ma13112598

**Published:** 2020-06-07

**Authors:** Hyunwoo Kim, Soon-Kook Hong, Jae-Kwan Ryu, Sung-Hoon Park

**Affiliations:** 1 Department of Mechanical Engineering, Soongsil University, 369 Sangdo-ro, Dongjak-Gu, Seoul 06978, Korea; hyunwoi@naver.com; 2 Department of Mechanical and Naval Architectural Engineering, Naval Academy, 1 Jungwon-ro, Jinhae-gu, Changwon-si, Kyungsangnam-do 440-749, Korea; hsk753@navy.mil.kr; 3 LIG Nex1, 207 Mabuk-ro, Giheung-gu, Yongin-si, Gyeonggi-do 16911, Korea; jaekwan.ryu@gmail.com

**Keywords:** carbon nanotube, polymer composite, aligned MWCNT, piezo-resistive characteristics

## Abstract

Highly aligned multi-walled carbon nanotube (MWCNT) polymer composites were fabricated via a roll-to-roll milling process; the alignment of the MWCNTs could be controlled by varying the speed of the rotating rolls. The effect of MWCNT alignment on the polymer matrix was morphologically observed and quantitatively characterized using polarized Raman spectroscopy. To provide a more detailed comparison, MWCNT composites with alignment in the transverse direction and random alignment were fabricated and tested. Enhanced mechanical and electrical properties were obtained for the aligned MWCNT composite, which can be attributed to the efficient electrical network and load transfer, respectively. In addition, a cyclic stretching test was conducted to evaluate the piezo-resistive characteristics of the aligned MWCNT composites. The composites with an aligned filler configuration showed an exceptionally high degree of strain sensitivity compared to the other composites.

## 1. Introduction

Carbon nanotubes (CNTs) have shown great potential for improving the performance of polymer matrixes when used as a filler, owing to their unique electrical [1,2,3], mechanical [4,5] and thermal [6,7,8] properties. Conductive CNT polymer composites demonstrate sensitive changes in electrical resistance under an applied external force, thus making ideal candidates for strain sensor applications [9,10,11,12]. Despite the development of various conductive composites, with carbon black and graphene as fillers [13,14], composites consisting of a one dimensional (1-D) filler with a high aspect ratio (such as CNT or silver wire) in an insulating polymer matrix have advantages regarding low electrical percolation and an efficient electrical network [15]. In previous studies, depending on the alignment direction of the CNTs, the physical properties of CNT composites, such as electrical conductivity, mechanical properties and strain-resistance sensitivity, could be changed [16,17,18,19]. Various attempts to form aligned CNTs have been successfully reported [9,10,20,21,22,23,24]. Kaushik et al. [10] produced an aligned CNT polycarbonate composite used as a strain sensor via a micro-injection method and observed its characteristics along the CNT-alignment direction. The direct writing technique of depositing a CNT composite on a substrate similar to a printer that extrudes ink on paper tends to align the CNTs in the extrusion direction during extrusion of the CNT composite due to the influence of shear flow [25]. Alternatively, Sohn et al. [6] fabricated aligned CNT polydimethylsiloxane (PDMS) composite films via roll-to-roll milling, discovering a phenomenon in which CNTs were partially aligned; they measured electrical conductivity along the aligned and transverse directions as a function of multi-walled CNT concentration and alignment direction.

In this study, we fabricated a highly aligned multi-walled CNT/PDMS composite film via a roll-to-roll milling process. The roll-to-roll milling method aligns the inner multi-walled carbon nanotubes (MWCNTs) by applying a circumferential shear to the uncured composite paste injected between the rolls, which rotate at different speeds; the degree of alignment can be adjusted by changing the roll speed. The resulting MWCNT composite with different degrees of alignment was compared with randomly oriented and transverse direction MWCNT composites. In order to characterize the MWCNT alignment in nanotube composites, polarized Raman spectroscopy was used for aligned MWCNTs, taking advantage of the relative intensity of the observed D and G band’s sensitivity to the nanotube’s orientation [5,20,21,26]. To investigate the effect of MWCNT filler alignment, mechanical tests and cyclic stretching tests were conducted for the three composite cases (aligned, random and transverse).

## 2. Materials and Methods

### 2.1. Materials

The silicone elastomer (Sylgard 184 A) and curing agent (Sylgard 184 B) were purchased from Dow Corning (Midland, MI, USA). The polydimethylsiloxane (PDMS) was formed by mixing the silicone elastomer and the curing agent at a ratio of 10:1. The MWCNT powder (JENO TUBE 6A, length = 50–150 μm, diameter = 5–7 nm) was purchased from JEIO (Incheon, Korea).

### 2.2. Fabrication of MWCNT/PDMS Composite

**Figure 1:**
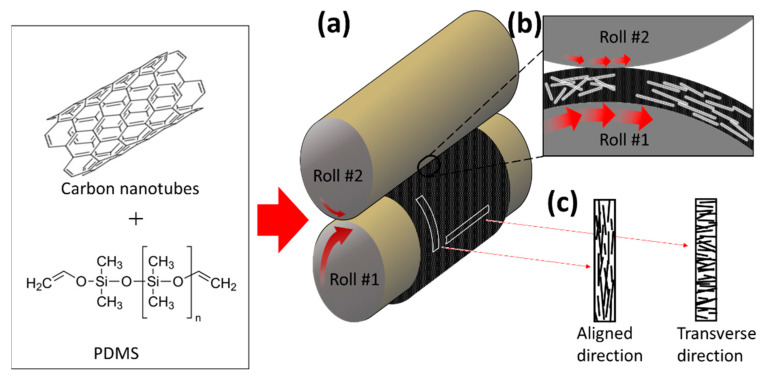
Fabrication scheme of the aligned multi-walled carbon nanotube (MWCNT)/PDMS composite. (**a**) Diagram of roll-to-roll milling. (**b**) The internal MWCNTs orientated in the circumferential direction, caused by shear force. (**c**) The aligned direction and transverse direction were obtained by cutting the film in the circumferential and axial directions, respectively.

To thoroughly disperse the highly entangled MWCNTs throughout the polymer matrix, 5 wt % of MWCNT and PDMS were premixed using a paste mixer for several minutes and subsequently three-roll milled for 10 min, while decreasing the gaps between the rolls. A roll-to-roll mill (tx-2053st-sp, intec, Gyeonggi-do, Korea) was constructed to fabricate an aligned MWCNT composite film. The diameter of both rolls was 56 mm and the roll gap was adjustable in increments of 10 μm. Before the paste was injected, a film tape was attached to the roll surface so that the paste on the surface could later be easily removed. When V_1_ (velocity of roll #1) was faster than V_2_ (velocity of roll #2), the paste injected between the rolls was attached to the surface of roll #1, forming a film. Rolls rotating at different speeds and directions apply a shear force to the paste, aligning the MWCNTs in the circumferential direction (Figure 1). Due to the nature of the roll-to-roll method, a relatively high shear force was applied to the surface compared to the inside of the film. To minimize this, a film was fabricated by injecting a specific quantity of paste while increasing the roll interval from 40 to 80 μm. Each process lasted 5 min, allowing sufficient alignment of the MWCNTs. The irregular pattern on the film surface resulting from roll-to-roll milling was an obstacle to the accurate analysis of the MWCNT’s alignment characteristics. Thus, after removing the film tape adhered to the roll surface, it was cured by applying light pressure (5 MPa) via a hot film-presser (Qmesys, Gyeonggi-do, Korea), at 150 °C for 1 h. A three-roll milled random array MWCNT/PDMS composite film and a pure PDMS film were fabricated by the same equipment under 15 MPa at 150 °C for 1 h, for use as the control group. In this study, to confirm the characteristics according to the degree of alignment of the MWCNTs, two types of composite films were fabricated with V_1_:V_2_ = 80:360 and 80:120 (RPM). For convenience, the ratio of V_2_/V_1_ is expressed as R_V_ (i.e., the ratio of the roll’s velocity). The thicknesses of the MWCNT/PDMS composite films made of R_V_ = 4.5 and 1.5 were both 450 μm, and the random array MWCNT/PDMS composite film was 500 μm.

### 2.3. Characterization

Nano/microstructure morphologies of MWCNT/PDMS composite films were characterized using SEM (GeminiSEM 300, Carl Zeiss Microscopy GmbH, Land Baden-Württemberg, Germany). The films were soaked in liquid nitrogen prior to cutting the cross-sectional areas. Polarized Raman spectroscopy, with an excitation wavelength of 514 nm, was performed to characterize the MWCNT alignment in the composite, using a Raman spectrometer (LabRam Aramis, Horiba Jobin Yvon, Kyoto, Japan). In the MWCNT-aligned complex, intensity data from the G and D peak was obtained when the MWCNT was parallel to the incident light and the scattered light was parallel (G_II_ and D_II_) and perpendicular (G_T_ and D_T_) to the incident light. Both results were obtained at the same location. For the random array MWCNT composite, data was collected at a fixed location in the same manner. During the tensile stretching and releasing cyclic tests, piezo-resistances were measured using a multimeter (DMM7510, Keithley, Cleveland, OH, USA) at room temperature. The tensile cyclic tests were performed at 20% of the original film length in the stretching direction, the number of cycles was 5 and the stretching and releasing speed was 20 mm/min. The length and width of the sample were 45 mm and 5 mm, respectively, and silver paste and copper tape were applied to both ends of the sample to minimize contact resistance. Mechanical properties were tested at room temperature using a universal tensile machine (DR-100, DRTECH, Seongnam, Korea) with a crosshead speed of 50 mm/min and the composite samples were prepared into dog-bone shapes.

## 3. Results and Discussion

**Figure 2:**
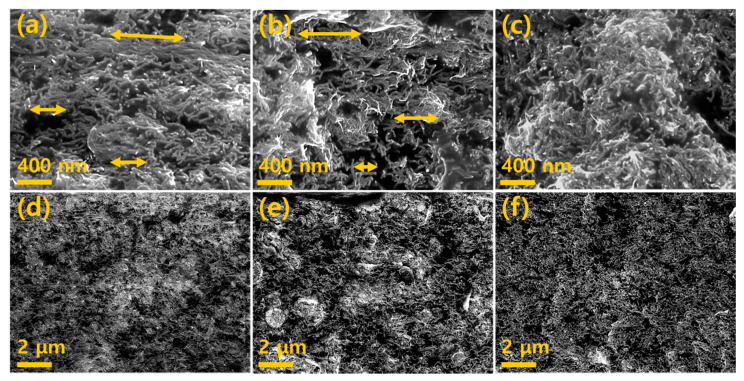
SEM cross-section images of: (**a**,**d**), aligned direction (R_V_ = 4.5). (**b**,**e**), aligned direction (R_V_ = 1.5). (**c**,**f**), random array MWCNT/PDMS composite film.

After the high viscosity MWCNT/PDMS composite paste (5 wt % MWCNT) was injected between two rotating rolls, the paste mostly formed on roll #1, which had a high roll speed, as shown in Figure 1a,b. MWCNT alignment in the composite occurred in the circumferential direction due to shear forces between roll #2 and the paste interface. The shear force was not quantitatively evaluated when uniform MWCNT alignment in the composite was achieved via roll-to-roll milling. However, as shown in Figure 2, the uniformly aligned MWCNT configuration in the composite and different degrees of alignment were confirmed by the R_V_ values. The higher the R_V_ value, the higher the degree of alignment of the MWCNTs. The aligned MWCNT composite film specimens cut in the circumferential and axial direction were referred to as aligned direction and transverse direction specimens, respectively (Figure 1).

### 3.1. Morphology and Raman Spectroscopy Analysis

SEM was used to confirm the morphology of the MWCNTs and MWCNT-dispersion inside the composite through a roll-to-roll milling process. Figure 2 shows the cross-section images of a MWCNT composite film formed at: R_V_ = 4.5, Figure 2a,d; R_V_ = 1.5, Figure 2b,e and random array, Figure 2c,f. It can be seen from the image of the random array sample that the MWCNTs were uniformly dispersed throughout the composite. As shown in Figure 2a, the MWCNTs were mostly aligned in the arrow direction; there is a significant difference compared to the randomly oriented MWCNTs shown in Figure 2c. MWCNTs at R_V_ = 1.5 have a smaller degree of alignment compared to those at R_V_ = 4.5, however, the alignment degree is high in comparison with the random array. Hence, it can be seen that the degree of alignment of MWCNTs increases as the R_V_ value increases.

**Figure 3:**
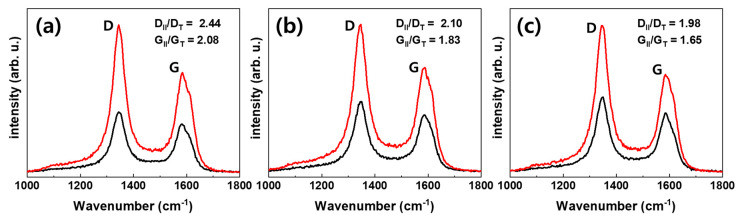
Raman spectra of multi-walled carbon nanotube (MWCNT)/PDMS composites; The red line measured at the incident light and the scattered light are parallel, and the black line measured at the incident light and the scattered light are perpendicular. (**a**) Aligned at R_V_ = 4.5, (**b**) aligned at R_V_ = 1.5 and (**c**) random array.

Polarized Raman spectroscopy was used to qualitatively analyze the level of alignment and the results are shown in Figure 3 and Table 1. The D band observed at 1350 cm^−1^ corresponds to disorders in the carbon atom’s bonds in CNTs and the G band observed at 1590 cm^−1^ corresponds to the graphitization degree of MWCNTs [21,27]. Increments in the D_II_/D_T_ and G_II_/G_T_ values correspond to the enhancement of MWCNT alignment in the polymer matrix [21,26,28]. As a result, G_II_/G_T_ increased from random, aligned (R_V_ = 1.5), and aligned (Rv = 4.5) to 1.65, 1.83 and 2.08, respectively, and D_II_/D_T_ increased to 1.98, 2.10 and 2.44, respectively. As R_V_ was increased, the alignment of the MWCNTs increased accordingly.

### 3.2. Mechanical Properties

**Figure 4:**
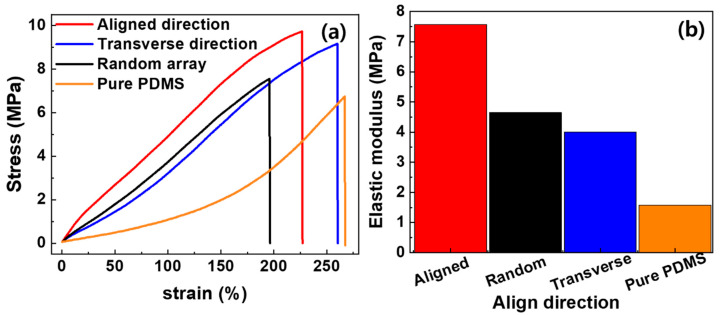
Mechanical properties of pure PDMS, randomly oriented, and aligned at R_V_ = 4.5 (aligned and transverse direction) MWCNT/PDMS composites; (**a**) stress–strain curve and (**b**) elastic modulus.

The mechanical properties depend on the load transfer capability between the MWCNTs and the matrix; an improved load transfer signifies enhanced mechanical properties. This can be achieved by aligning the CNTs [4,5,20,29]. We investigated the effects on mechanical properties of aligning MWCNTs in a polymer matrix. The analysis was conducted on the aligned MWCNT composite films, fabricated by roll-to-roll milling. The tensile stress–strain results of the MWCNT/PDMS composites along the aligned, transverse and random directions and pure PDMS are shown in Figure 4, where a load was applied until the specimen fractured. It was confirmed that the addition of 5 wt % MWCNTs to PDMS, even without alignment, resulted in an improvement to the Young’s modulus and tensile strength by 194% and 12%, respectively, compared to pure PDMS. The aligned direction recorded an improvement to the Young’s modulus and tensile strength by 63% and 28%, respectively, compared to the random array.

However, the transverse direction recorded a reduction in Young’s modulus by 14% and an improvement to the tensile strength by 21%, compared to the random array.

The MWCNTs aligned parallel to the tensile direction showed an efficient load transfer across the MWCNT–matrix interface as the composite was stretched, with a corresponding increase in the elastic modulus. As large tensile deformation occurs, the nanotubes act as a bridge to the micro-cracks generated in the matrix, changing the crack direction and delaying fracture, thereby improving elongation [30,31]. For MWCNTs aligned perpendicular to the tensile direction, the initial tension showed poor load transfer across the MWCNT–matrix interface, however, as a larger strain was applied in the tensile direction the MWCNT also partially aligned along the tensile direction; eventually improving the extensibility [32]. Random array specimens showed higher tensile stress than transverse direction specimens, for the same strain, as more MWCNTs were parallel to the tensile direction, resulting in higher load transfer. However, the random array specimen, in which MWCNTs were entangled together, had limited alignment by tensile deformation and showed lower tensile strength than the transverse direction specimen.

### 3.3. Electrical Properties

Inserting a CNT filler into a polymer matrix can effectively improve conductivity [7,33]. In addition, aligned CNTs can provide higher electrical conductivity compared to randomly oriented CNTs [19]. Studies have reported that the electrical properties of CNTs improve with higher aspect ratios and content [15]. Roll-to-roll milling for the alignment of MWCNTs in a composite can be an effective method for demonstrating the degree of alignment due to shear forces. The electrical conductivity of MWCNT/PDMS composites along the aligned and transverse direction, fabricated by roll-to-roll milling, and random array MWCNT/PDMS composites are shown in Table 1. The electrical conductivity of random, aligned (R_V_ = 1.5) and aligned (R_V_ = 4.5) were 88, 92 and 101 S/m, respectively, and the random, transverse (R_V_ = 1.5), and transverse (R_V_ = 4.5) were 88, 67 and 57 S/m, respectively. In the case of the MWCNT/PDMS composite along the aligned direction, MWCNTs were mostly positioned parallel to the direction of the applied current, forming the most electrical networks, thus the electrical conductivity increased [11]. Furthermore, this tendency is clearly shown as the degree of alignment increased. Conversely, the transverse composite, in which the MWCNT was positioned perpendicular to the direction of the applied current, contained the smallest number of electrical networks; this resulted in decreased conductivity. The measured electrical conductivities are listed in Table 1.

### 3.4. Piezo-Resistive Properties

**Figure 5:**
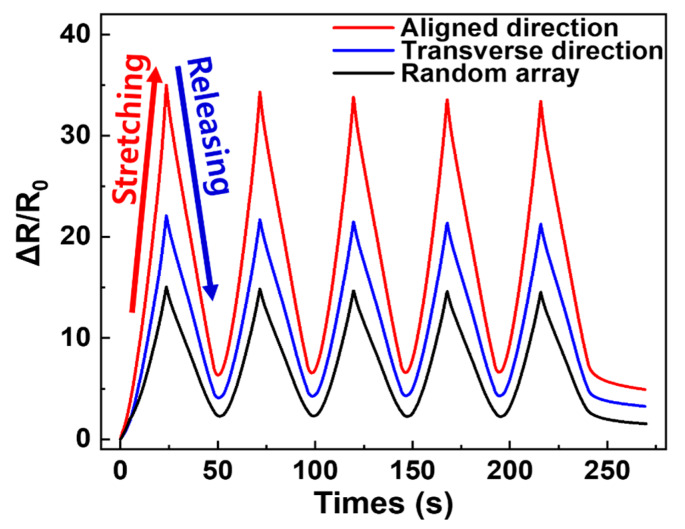
Piezo-resistive characteristic during stretching and releasing cyclic test from 0 to 20% strain for random, aligned at R_V_ = 4.5 (aligned and transverse direction) MWCNT/PDMS composites. A 40 mm strain sensor repeated at the speed of 20 mm/min.

**Figure 6:**
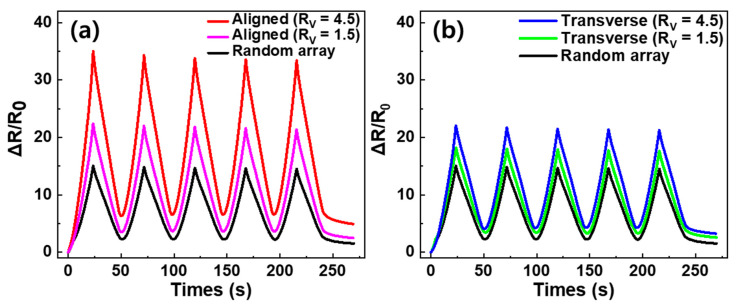
Comparison of CNT/PDMS composite by the degree of alignment. Cyclic test under tensile strains applied from 0 to 20% of the (**a**) aligned direction and (**b**) transverse direction.

Piezo-resistive properties are important for obtaining information about the electrical network coupling of MWCNTs; different changes in resistance can be observed by applying an external force to the specimen. We investigated the piezo-resistance of the aligned and transverse direction composites, fabricated via a roll-to-roll milling method and random array of the MWCNT networks in a polymer matrix; this was to determine the performance as a strain sensor. Figure 5 shows the variation in resistance during five cyclic stretching tests, applying 20% tensile strain to three differently oriented specimens. After the first stretching process, ΔR/R_0_ was 35%, 22% and 15% in the aligned direction, transverse direction and random array, respectively. The order of these results did not match the order of electrical conductivity. To explain the resistance change during stretching, Jin et al. [34] reported that when randomly oriented CNT composite film is stretched, the CNTs reorient in the direction of stretching and slide past each other. Thus, during the tensile process it can be assumed that applying tension to the random array MWCNT/PDMS composite film partially reorients the direction of the MWCNTs in the stretching direction and creates new contact points, such that the percent of change in resistance is small. Similar to the random array sample, stretching the transverse direction composite film partially reorients the MWCNTs into the stretched direction, which induces the creation of new contacts. However, in the transverse direction composite film there are fundamentally a small number of networks and many contacts in the electrical path. Therefore, it is reasonable to assume that increasing the contact distance of MWCNTs involves a large change in resistance. Furthermore, in the process of stretching the aligned direction MWCNT composite, the specimen showed the greatest change in resistance, with the small possibility of creating new contacts as the MWCNT contact distance increased. Subsequent to the first releasing process of all specimens, ΔR/R_0_ decreased due to the reconnection between MWCNTs and the relocation of the electrically conductive path. It was observed that the initial values of ΔR/R_0_ could not be fully recovered after the first release process for each applied strain; this is due to the hysteresis effect caused by the viscoelastic matrix and permanent damage to the electrically conductive network [35]. Hysteresis confirms that the loss of the permanent network was significant in the aligned, transverse and random directions, with ΔR/R_0_ = 6.4%, 4.1% and 2.3%, respectively. After the first stretch-release cycle, the composite specimens demonstrated a stable sensing signal. Figure 6 shows the difference in ΔR/R_0_ with R_V_ for the specimens. For the aligned direction MWCNT/PDMS composites, the tensile-ΔR/R_0_ increased as R_V_ increased, as shown in Figure 6a. After the initial stretching cycle, the random array, R_V_ = 1.5 and R_V_ = 4.5 samples had a change in resistance of 15%, 22% and 35%, respectively. Similarly, for the transverse direction MWCNT/PDMS composites the random array, R_V_ = 1.5 and R_V_ = 4.5 samples had a change in resistance of 15%, 18% and 22%, respectively. It can be seen that the piezo-resistance increased as the degree of alignment increased. Table 2 shows the various attempts to align MWCNTs in the composite with mechanical, electrical and piezo-resistive characteristics. Results showing similar trends to our results are marked with green shaded cells. The tendency for electrical conductivity was consistent with the results we reported, while mechanical properties and resistance change during tensile test were often different. The difference could be attributed to interaction between MWCNT and the polymer matrix, the type of polymer, the content of filler and the manufacturing method.

## 4. Conclusions

We confirmed that the degree and direction of alignment MWCNTs in the polymer composite affects the composite’s mechanical, electrical and electro-mechanical properties. It has been demonstrated that roll-to-roll milling can significantly influence the alignment of MWCNTs in a polymer matrix. SEM images, D_II_/D_T_ and G_II_/G_T_ from polarized Raman spectroscopy showed that as R_V_ increased, the alignment of the MWCNTs increased, which was attributed to the applied shear forces. The electrical conductivity, Young’s modulus, tensile strength and strain sensitivity were highest for the aligned direction MWCNT/PDMS composite due to the effective load transfer and conductive network of MWCNTs. On the contrary, the electrical conductivity and Young’s modulus were lowest for the transverse direction MWCNT/PDMS composite.

## Figures and Tables

**Table 1:** Electrical/mechanical properties and Raman intensity ratio of the CNT/PDMS composites and pure PDMS.

Align Direction	R_V_ (V_1_/V_2_)	Electrical Conductivity [S/m]	Tensile Strength [MPa]	Young’s Modulus [MPa]	Raman Intensity Ratio Parallel/Perpendicular	
					G_II_/G_T_	D_II_/D_T_
Aligned	4.5	101	9.72	7.57	2.08	2.44
	1.5	92	8.53	5.12	1.83	2.10
Transverse	4.5	57	9.16	4.00	-	-
	1.5	67	8.77	4.09	-	-
Random	-	88	7.55	4.65	1.65	1.98
Pure PDMS	-	-	6.72	1.58	-	-

**Table 2:** Electrical, mechanical properties and piezo-resistive characteristic during tensile stress of CNT/polymer based composites fabricated by various methods.

Reference and Year	Align Direction	Electrical Conductivity (S/m)	Tensile Strength (MPa)	Young’s Modulus (MPa)	Resistance Change (%)	Fabrication Method	Type of Composite
Current study	Aligned direction	101 (5 wt %)	9.72	7.57	35.0 (0 to 10%)	Roll-to-roll mill, hot film-presser	5 wt % MWCNT/PDMS composite
	Transverse direction	57 (5 wt %)	9.16	4.00	21.9 (0 to 10%)		
	Random array	88 (5 wt %)	7.55	4.65	14.9 (0 to 10%)	hot film-presser	
[10], 2013	Aligned direction	R_0_ = 13.9 kΩ	-	-	ΔR/ΔL = 3.65	Injection molding	5 wt % MWCNT/poly-carbonate composite
	Transverse direction	R_0_ = 39.9 kΩ	-	-	ΔR/ΔL = 6.5		
[21], 2018	Aligned direction	3 × 10^−4^	-	5500	-	DC electrical fields applied	0.5 wt % MWCNT/epoxy resin composite
	Transverse direction	9 × 10^−5^	-	-	-		
	Random array	6 × 10^−5^	-	3900	-		
[11], 2016	Aligned direction	8 × 10^−5^	34	1200	-	DC electrical fields applied	MWCNT/poly vinylidene fluoride composite
	Transverse direction	1.5 × 10^−5^	39	1210	-		
	Random array	1.8 × 10^−5^	26	950	-		
[4], 2015	Aligned direction	5.25 × 10^−3^	72	1680	-	DC electrical fields applied	0.5 wt % MWCNT/chito-san composite
	Transverse direction	4.57 × 10^−8^	50	1620	-		
	Random array	4.77 × 10^−3^	68	1640	-		
[12], 2018	Aligned direction	R_0_ = 4.96 kΩ	9.5	4.7	9	CNT film on the PDMS	
	Transverse direction	R_0_ = 69.6 kΩ	7.5	4.4	1		
